# Status and future prospects of biomedical engineering: a Japanese perspective

**DOI:** 10.2349/biij.3.3.e37

**Published:** 2007-07-01

**Authors:** M Kikuchi

**Affiliations:** Department of Engineering, National Defense Medical College, Japan

## INTRODUCTION

**Figure 1:**
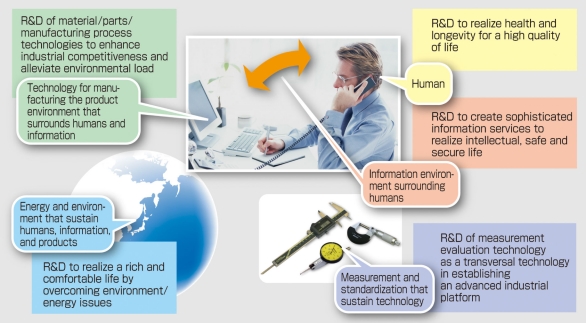
R&D to realise a society of sustainable development (from AIST’s “Second Period Research Strategy”).

The 21^st^ century is considered the “human” century, the underlying reason of which is the increasingly serious issue of declining birth rate and aging population shared by advanced nations. By the latter half of this century, countries bearing large populations such as China and India will also eventually begin to experience aging of their populations. Therefore, the development of technologies related to medical care, health, and welfare are considered extremely crucial. In addition to expectations for the development of medical technologies arising from needs of the global economy, there are also increasing expectations for the creation of new medical technologies stemming from trends in development and practical application of the life sciences. AIST’s Second Period Research Strategy of the AIST (National Institute of Advanced Industrial Science and Technology) in Japan established in April 2006 also clearly states that developments in science and technology focusing on humans will become the mainstream future trend (Figure 1). Furthermore, a strategic target has been set in the field of health with regards to research strategies for the area of life sciences.

**Figure 2:**
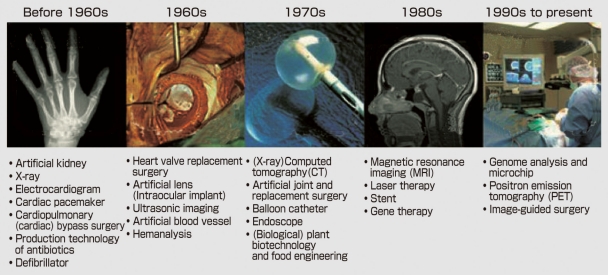
Great Medical equipment achievements elected by AIMBE to have most contributed to clinical medicine over the past 50 years (AIMBE).

At the beginning of last year, AIMBE (American Institute for Medical and Biological Engineering) conducted a survey for the purpose of assessing the role that medical equipment and technology has played in clinical medicine since the 1950s. The medical equipment that contributed most to clinical medicine/medical care in the 20^th^ century were chosen for each ten-year period (Figure 2). The list contained equipment/technologies that anybody would agree upon, clearly showing significant support of medical instruments in the development of leading-edge medical care.

Development of biomedical instrumentation technology to measure the physical and chemical information and signals emitted by the living body in a minimally (or non-) invasive and continuous manner, was responsible for establishment of the concept of patient monitoring within clinical medicine. It thus contributed greatly to “enhancement of the quality of medical care.” Furthermore, the technology such as medical imaging evolved into two-dimensional from one-dimensional measurements, and was further developed into three-dimensional measurements, and made speedier as well. Now, it is not an overstatement to say that medical imaging has entered four-dimensional measurement. Meanwhile, treatment technologies have also progressed from highly invasive operative treatments to minimally invasive techniques such as endoscopic surgery. At the end of the 20^th^ century, the so-called grand compilation of technologies such as image-guided computer-assisted surgery and robotic surgery, has begun and is being used in clinical application. In the future, extremely delicate and precise microscopic surgery that cannot be performed by a surgeon can be possible through the combination of new surgery support systems and the surgeon’s skill. Thus, progresses in medical treatment technologies simultaneously promote the development of medicine itself. Many new medical findings, such as the progress in anesthesiology required in awake surgery, are appearing in rapid succession. Progress in biomedical engineering in the 20^th^ century has greatly transformed the concept of health care itself, by transfiguring it dramatically from a form of “classical medical care” to that of “leading edge medical care assisted by instrumentation and imaging.” Furthermore, social demands to “enhance and maintain a high quality of medical treatment, health and welfare” in this aging society are behind the anticipation for further developments in this field in the future.

The United States established AIMBE with the assistance of the National Science Foundation (NSF) and others, in February 1992, with the following foresights. First, the focus of medical technology will shift from diagnostic to treatment technology; progresses in molecular biology will bring genetic engineering into diagnostic technology; micro- and nano-treatment technologies will be developed as the ultimate in minimally invasive endoscopic treatment technology; and further, regenerative medicine, the ultimate treatment method, will become possible in the near future through tissue engineering. In AIMBE, many academic societies involved in research and development of new medical treatment technologies of the 21^st^ century have united in a common purpose and have been active thus. Later, in 2001, the National Institute for Bioimaging and Bioengineering (NIBIB) was founded within the National Institutes of Health (NIH), based upon the foundation of the Bioengineering Consortium (BECON). In accordance with this concept for BECON in USA, Japan also established the Medical Engineering Technology Industrial Strategy (METIS) consortium in 2001.

**Figure 3:**
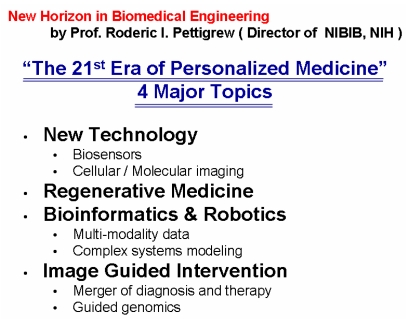
Direction of research and development of medical equipment targeted by NIBIB for the 21^st^ century.

Figure 3shows the target research topics set forth by NIBIB’s first director, Dr. Roderic I. Pettigrew. These topics show that movements in 21^st^ century medical technology are based upon nanomedicine, regenerative medicine and genetic medicine.

As analysis of the human genome is virtually complete, medical technology will now evolve into a technology where various interdisciplinary technologies are multiply combined, from the level of molecules and genes below 100 nm, to tissue and organs, and further to virtual human science per individual level. In addition to the technology of mass cell culture, the validation technology for assuring the quality of the cells and tissue produced is an essential element in allowing medical technology employing tissue engineering to mature into a clinical technology. Thus the development of minimally invasive measurement techniques for regenerative medicine is anticipated. Molecular imaging technology is of growing interest with relevance to the genetic treatment technology. Molecular imaging is of potential in enabling direct observation of function expression of genes in genetic treatments, while various molecular imaging techniques are also anticipated for other function-specific imaging. In clinical medicine, the structure and function of the protein produced by the gene is more often linked directly to the disease rather than the genetic information itself. The proteome is attracting post-genome attention, and it requires a method of analysis different from that of the genome. The protein chip is also being developed as a new technique.

## BIOMEDICAL ENGINEERING TECHNOLOGY AND IMPROVEMENT IN THE QUALITY OF MEDICAL TREATMENT

**Figure 4:**
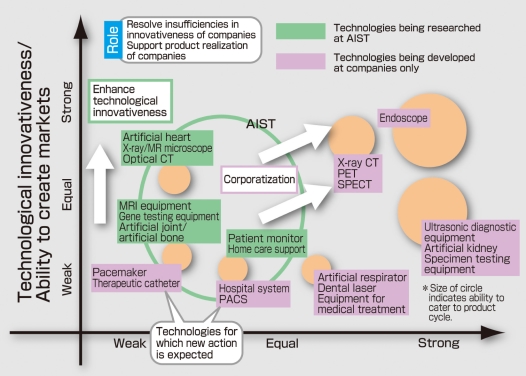
The competitiveness of Japan’s medical equipment industry and AIST’s role (From AIST’s “Second Period Research Strategy”).

Endoscopy may be cited as a typical example of minimally invasive instrumentation technology. In 1950, the gastrocamera was developed in Japan for the first time in the world. Then the prototype of the present-day endoscope was developed in 1957, consisting of a fiberscope endoscope for extracorporeal observation and imaging. Later, the electronic endoscope consisting of an endoscope equipped with a CCD camera on its tip was developed. Furthermore, the capsule endoscope was developed in the beginning of the 21^st^ century. The wireless capsule endoscope manufactured by Israel’s Given Imaging Ltd. was approved in Europe in May and by the U.S. FDA in August of 2001. The small intestine is a digestive tract with a total length of 7-8 m, and unlike the large intestine, it is virtually unfixed within the body cavity. It has been referred to as “the last Dark Continent” in gastroenterology. Now, however, the time has come for endoscopic technology to demonstrate the effect in finally allowing observation of the entire digestive tract. Meanwhile, endoscopic systems are used not only in the diagnosis of diseased regions, but are also demonstrating great effect in treatments, such as gastric polyp snare loop techniques, which began from biopsy under direct vision by fiberscope starting in 1965, laser hemostatic techniques which followed, as well as in resections of the gastric mucous membrane. Hence, history shows that endoscopic technology is an area in which Japan leads the world. At the same time, it has technical innovation and market creation ability, as well as competitiveness of product development against Europe and U.S., as seen in Figure 4, offering bright prospects to Japan’s medical equipment industry from both of these sides.

In addition, in the future of diagnostic endoscopy, this author hopes to see alleviation of the discomfort experienced upon insertion and during observation (by using capsule endoscopes and virtual endoscopy, etc.) as well as improvements in diagnostic capabilities (through ultra-magnifying endoscopes, confocal laser microscope endoscopes, and optobiopsies, which make on-the-spot “virtual biopsies” and “virtual pathologic examinations” possible, requiring no biopsy, using an endoscope equipped with an ultra-compact spectroscope with MEMS on its tip among others). Meanwhile in treatment endoscopy, extended applications of new minimally invasive endoscopic surgical treatments can be expected, such as in endoscopic fetal surgery and endoscopic brain surgery, as well as further coordination with navigation technologies and surgery support systems to allow safe execution of these procedures.

In addition, various developments are anticipated in endoscopic treatment techniques, from the conventional treatments centering on physical energies, to fusion with drug delivery systems as in the case of the drug-eluting stent, as well as endoscopic systems fused with cell and genetic treatments, to be discussed later.

## MEDICAL IMAGING TECHNOLOGY AND SUSTAINING LEADING EDGE MEDICAL TREATMENT

About 30 years ago, practical application of the X-ray CT made possible tomography of the human brain, thus revolutionising the diagnosis of neurological disorders. In addition, about 20 years ago, MRI was made clinically applicable, and the technological progress to follow allowed for revolutionary developments in diagnostic imaging of the total body. Although diagnostic imaging and molecular biology are examples of the technologies/disciplines that brought about significant reform to modern medicine, today, they are attracting attention in the further evolved form of molecular imaging. The morphological diagnostic methods of X-ray CT and MRI are already beneficial for functional diagnosis, while SPECT and PET are even becoming established as methods for metabolism diagnosis. As developments in functional MRI and PET have enabled for the understanding of higher brain functions and to conduct molecular function imaging and molecular imaging, they are hoped to serve as the platform technology for genetic treatment evaluation methods and new drug discoveries.

The objective of molecular imaging is to make visible the activities of the living body on a molecular level over time by commanding various imaging methods, to thereby understand the significance of molecular mechanisms in life science. The information thus obtained is used not only to understand molecular mechanisms, but may be used also as the basic data for diagnosis. It may potentially provide findings to become the seeds for development of new future medicine, such as drug developments and application to drug delivery techniques. Molecular imaging is regarded as a “specific function imaging,” thus targets for realising imaging, which are currently being tried, include neurotransmission/receptor imaging, antibody/peptide imaging, and also a genetic imaging technique using antisense and reporter genes (genes used to confirm whether or not the gene has actually entered the target region by embedding genes of proteins or receptors that can be imaged near the target gene for treatment).

“Molecular imaging” is evolving in two directions: (1) visualisation targeting specific molecules in the living body, and (2) visualisation on a microscale, in the range of cells. Future technical development and research needs to be promoted in each of these directions. In addition, dynamic time-course visualisation in the form of “molecular dynamic imaging” is also an important element.

The first direction stated above is a field best performed by conventional nuclear medical equipment (PET in particular), thus future issues will most likely be dominated not by imaging techniques but rather by the development of drugs such as tracers. Meanwhile, the second direction above requires technical developments in the imaging technology itself. New magnetic resonance spectroscopic imaging (MRSI) and other new imaging methods are anticipated to replace the conventional MRI. In addition, molecular imaging will become extremely important as the validation technology for regenerative medicine and cell therapy, which will lead to progress of great importance to medicine in the future.

Immediate issues in molecular imaging include the following:

1. Molecular imaging is important in many aspects of cancer, its largest target. Presently, ^18^F-labeled deoxyglucose (FDG), which is also used in humans, is becoming the gold standard in cancer diagnosis, although various other techniques are also being explored. In the future, the knowledge from cell biology needs to be extended to imaging technology of the entire body, and to develop new imaging agents as well. In the metastasis of cancer cells, the relation of *in vivo* inter-cell bonding is not well understood, but the mechanism of metastasis and development of cancer is expected to be elucidated through imaging.
2. Molecular imaging is important in molecular and gene therapies, where the reporter gene must be used effectively. There are two techniques for molecular imaging using gene expression: antisense therapy and a method using the reporter gene.
3. By uniting the research of genes and new receptors with imaging technologies, then fusing together various methods of imaging, it becomes possible to image the dynamic processes within the living body. Then, by further applying such information, it is more likely to become applicable to diagnosis and assessment of various illnesses, and is expected to allow easy viewing of the distribution of drugs used in treatment.
4. Optical technology, and the technology for utilisation of near infrared rays in particular is recently making remarkable progress. The possibility for light from substances within the body to be imaged by tomography using this technology allows various things to be employed as reporter genes. Continuous changes can then be observed for prolonged periods using optical technology, unlike the case with PET which is not practical as it causes exposure.

As described above, in molecular imaging, the knowledge of molecular biology and also interfaces with various engineering technologies are crucial. Thus, in addition to the collaboration between medicine, engineering and industry advocated nowadays, further coordination with medicine and biology is also required.

## THE ROLE OF BIOMEDICAL ENGINEERING IN THE FUTURE

As illustrated above, medical technology has been experiencing significant transformations since entering the 21^st^ century. In addition to conventional technologies, biotechnology and nanotechnology made applicable are being fused anew into medical technology. The advanced health care that citizens benefit from every day is realised upon the latest medical knowledge and equipment/technology. Medical equipment has, in the past, shown progress every ten years. Computerisation and systemisation of measuring and monitoring equipment for biophenomena was implemented in the 1960s; various medical imaging technologies were born in the 1970s; minimally invasive endoscopic diagnosis and treatment technologies appeared in the 1980s; and robotic surgery was realised in the 1990s. An extremely interesting fact is the Japanese medical system also underwent radical transformations in sync with these stages. In the 1960s, it was “scale-up of the volume of health care” by establishing more medical facilities; in the 1970s, it was “improvement in the quality of health care”; in the 1980s, it was “the issue of medical costs”; and finally in the 1990s, it was “the balance between cost and quality of health care.” The nature of Japan’s health care has actually been affected directly by the progress in, and supply of, medical equipment and technologies. The fact constitutes a typical example of the coordination between technical development and the social system, suggesting that developments in medical technologies are not merely limited to the realm of scientific research.

**Figure 5:**
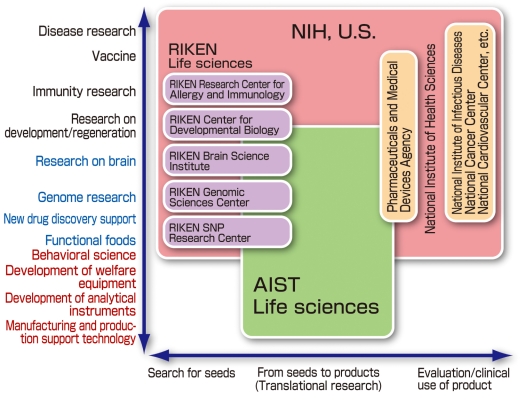
Biomedical Engineering covered by various worldwide institutions in areas of genome, medicine and health (From AIST’s “Second Period Research Strategy”).

A stable supply of advanced health care quite simply represents the needs of the public and society. In the future, “translational research” needs to be urgently constructed and should consist of “basic medicine and engineering research” such as the combination of bio- and nanotechnologies and further augmented with new knowledge. These outcomes are then used to actually implement Product Realisation Research for supply to the clinical medicine. Figure 5is an outline of the range of activities covered by NIH, the world-leading U.S. research institute, in the fields of genome, medicine, and health, as well as Japan’s RIKEN, various research institutes of the Ministry of Health, Labour and Welfare, and government institutions. AIST’s life sciences-related research units are required to engage in basic research from an industrial perspective not covered by any of the above, namely “the phase of searching for seeds and actively linking them to products, and the scientific evaluation of product functions.” AIST thereby plays an indispensable function in promoting medical equipment industry.

